# Cost-Effectiveness of Cryopreserved vs Liquid-Stored Platelets for Managing Surgical Bleeding

**DOI:** 10.1001/jamanetworkopen.2025.54363

**Published:** 2025-12-08

**Authors:** Zhomart Orman, Michael C. Reade, Denese C. Marks, Belinda D. Howe, Michael Bailey, Alayna Carrandi, Zoe K. McQuilten, Alisa M. Higgins

**Affiliations:** 1 Australian and New Zealand Intensive Care Research Centre, School of Public Health and Preventive Medicine, Monash University, Melbourne, Victoria, Australia; 2 Medical School, University of Queensland, Brisbane, Queensland, Australia; 3 Joint Health Command, Australian Defence Force, Canberra, Australian Capital Territory, Australia; 4 Research and Development, Australian Red Cross Lifeblood, Sydney, New South Wales, Australia; 5 Transfusion Research Unit, School of Public Health and Preventive Medicine, Monash University, Melbourne, Victoria, Australia

## Abstract

**Importance:**

Platelet transfusion is critical in managing major bleeding, but access to liquid-stored platelets is limited by their 5- to 7-day shelf life. Cryopreserved platelets last up to 2 years and may improve availability, although their cost-effectiveness remains uncertain.

**Objective:**

To evaluate the cost-effectiveness of cryopreserved platelets vs liquid-stored platelets for managing surgical bleeding.

**Design, Setting, and Participants:**

An economic evaluation was conducted from the Australian government funder perspective alongside the CLIP-II randomized clinical trial, following intention-to-treat principles. Adult patients (age ≥18 years) undergoing cardiac surgery at 11 Australian tertiary hospitals between August 2021 and April 2024 were screened for eligibility. Eligible patients were those at high risk for platelet transfusion, identified by risk prediction score or clinician judgment, excluding female patients aged 18 to 55 years who were rhesus D (RhD) negative or with unknown RhD status and patients with coagulopathy or prior thromboembolism. Data were analyzed between January and April 2025.

**Interventions:**

Participants received at least 3 units of either cryopreserved or liquid-stored platelets intraoperatively or within 24 hours postsurgery.

**Main Outcomes and Measures:**

Costs were estimated in 2023 Australian dollars (A$) by assigning unit costs to patient-level resource use in each trial group, including intensive care unit (ICU) admissions, hospitalizations, and blood products. Effectiveness measures included postoperative bleeding volume (first 24 hours post-ICU admission and total), 90-day mortality, type 4 bleeding (based on the Bleeding Academic Research Consortium criteria), and serious adverse events. Given the 90-day follow-up period, discounting was not applied.

**Results:**

Among 202 participants (median [IQR] age, 66 [57-74] years, 153 [75.7%] male), 104 received cryopreserved platelets and 98 received liquid-stored platelets. The mean difference in per-patient cost was A$15 035 (95% CI, −A$1878 to A$31 949) with cryopreserved platelets vs liquid-stored platelets. Mean blood loss within 24 hours following ICU admission was 121 (95% CI, 5 to 237) mL higher, total postoperative blood loss was 504 (95% CI, 145 to 862) mL higher, and type 4 bleeding was 12.3% (95% CI, 1% to 23.6%) higher with cryopreserved platelets compared with liquid-stored platelets. The difference in 90-day mortality between cryopreserved platelet and liquid-stored platelet groups was not statistically significant (7.4% [95% CI, −0.3% to 15.1%]). Only 1 serious adverse event was observed in each group.

**Conclusions and Relevance:**

In this economic evaluation, cryopreserved platelets were dominated by liquid-stored platelets for managing bleeding in cardiac surgery. Further research is needed to assess the potential economic benefits of cryopreserved platelets in broader populations, particularly in regional and remote hospitals where platelet availability is limited.

## Key Points

**Question:**

Are cryopreserved platelets cost-effective compared with liquid-stored platelets for managing active bleeding in patients undergoing cardiac surgery at high risk of platelet transfusion at Australian tertiary hospitals?

**Findings:**

In this economic evaluation including 202 adults enrolled in a randomized clinical trial, the mean difference in per-patient cost was A$15 035 (95% CI, −A$1878 to A$31 949) with cryopreserved platelets compared with liquid-stored platelets, although the difference was not significant. Mean blood loss within 24 hours after intensive care unit admission was 121 (95% CI, 5 to 237) mL higher with cryopreserved platelets, a significant difference.

**Meaning:**

These findings suggest that treatment with cryopreserved platelets was more costly and less effective than liquid-stored platelets; therefore, liquid-stored platelets were preferred in cardiac surgery.

## Teaser

This economic evaluation examines the cost-effectiveness of cryopreserved platelets vs liquid-stored platelets for managing surgical bleeding in patients undergoing cardiac surgery.

## Introduction

Platelet transfusion is a crucial, lifesaving treatment for managing major bleeding. However, commonly used liquid-stored platelets stored under standard blood banking conditions (20 °C to 24 °C), have a short shelf life of 5 to 7 days, due to bacterial proliferation.^1^ Consequently, patients in rural, smaller hospitals or military field hospitals often have limited or no access to urgent platelet transfusions.^2^ Freezing platelets at −80 °C with a cryoprotectant (dimethyl sulfoxide) extends their shelf life to at least 2 years,^3^ reduces the risk of infectious disease transmission, and may enhance the hemostatic activity of platelets.^4,5^ This cryopreservation approach presents a promising solution for hospitals facing restricted access to platelets while alleviating the burden on blood banks.

Cryopreserved platelets were recently compared with liquid-stored platelets in adult patients undergoing cardiac surgery at high risk of platelet transfusion in the CLIP-II trial.^6^ However, the cost-effectiveness of cryopreserved platelets remains unknown. In this study, we assessed the cost-effectiveness of cryopreserved platelets compared with liquid-stored platelets alongside the CLIP-II trial for the management of cardiac surgical bleeding.

## Methods

This economic evaluation was conducted as part of the CLIP-II trial, which was approved by the Human Research and Ethics Committees of Austin Hospital, Melbourne, and the Australian Red Cross Lifeblood. Written informed consent was obtained from all participants prior to their enrollment in the study. Reporting of this study followed the Consolidated Health Economic Evaluation Reporting Standards (CHEERS) reporting guideline.^7^

This was a trial-based cost-effectiveness analysis comparing transfusion with cryopreserved platelets and liquid-stored platelets, as prespecified in the CLIP-II trial protocol^8^ and health economic analysis plan.^9^ A full economic evaluation was conducted, adhering to intention-to-treat principles and adopting a health care system perspective focused on inpatient services from the Australian government funder. As the follow-up was limited to 90 days, discounting of costs and benefits was not applied.

### Study Design and Participants

CLIP-II was a multicenter, blinded, randomized clinical trial (RCT) conducted in Australian metropolitan hospitals to evaluate the efficacy and safety of cryopreserved platelets compared with liquid-stored platelets in patients undergoing cardiac surgery.^8^ Adults aged at least 18 years who were identified preoperatively as being at high risk of requiring platelet transfusion were eligible for inclusion. High-risk status was defined as a score of 1 or greater on the Adult Cardiac Surgery Platelet Transfusion risk prediction tool^10^ or determined by clinical judgment. The primary outcome was the volume of bleeding within the first 24 hours following admission to the intensive care unit (ICU) after surgery. Further details regarding trial methods are available in the CLIP-II trial protocol^8^ and in the CLIP-II primary report.^6^

### Cost Estimation

Costs were estimated in 2023 Australian dollars (A$), equivalent to US $0.73 per A$1, based on the 2023 purchasing power parity conversion factor.^11^ Unit costs were then applied to quantities of resource use identified and measured in each trial group using patient-level data collected on case report forms (CRFs).^8^ Health care resources were measured from ICU admission after surgery up to 90 days or death, whichever occurred sooner, based on the length of the index ICU stay, index hospitalization (measured excluding all index ICU stays), ICU readmissions during the index hospitalization, hospital readmissions, inpatient rehabilitation, and blood products received.

The index ICU stay was categorized by the number of organs supported each day, based on the CRF data, including invasive mechanical ventilation, kidney replacement therapy, and extracorporeal membrane oxygenation. Since stratified ICU costs by the number of organs supported were unavailable in Australia, data from the UK National Cost Collection were used to estimate ICU admission costs.^12^ Weighted factors were calculated by dividing the mean daily ICU cost across all 5 organ support categories by the cost for each category. These weighted factors were then applied to the mean cost per ICU bed-day in Australia (A$6125 in 2022-2023, adjusted from A$4375 in 2013^13^ using the Consumer Price Index [CPI] in Health from the Australian Bureau of Statistics).^14^ This approach was used to estimate the cost of the index ICU stay based on the number of organs supported each day (eTable 1 in Supplement 1). The costs of subsequent ICU readmissions were calculated using the same mean daily rate, adjusted from 2013 values.^13^

The costs of index hospitalizations and hospital readmissions were estimated using an inflation-adjusted 2022 to 2023 national mean cost of A$2499 per bed-day. Because admission-level diagnostic codes were not collected in the CLIP-II trial, diagnosis-specific hospital costing was not possible. Instead, we applied the 2021 to 2022 national mean cost per acute admission (A$5808.5), inflated it to 2022 to 2023 values using the ratio of health CPIs (December 2022 to December 2023), and divided this by the national mean length of stay (LOS) of 2.44 days in public acute hospitals to derive a cost per bed-day.^15^ This approach is consistent with standard methods when only length of stay data are available. Additionally, the cost of rehabilitation was calculated based on the 2021 to 2022 mean cost per subacute cardiac separation, adjusted to 2022 to 2023, and estimated at A$19 675.^15^

Although drugs and fluids account for only 5% of ICU costs and are typically included in overall ICU expenses,^13^ the use of platelets, red blood cells (RBC), fresh frozen plasma, and cryoprecipitate in this study was measured separately using CRFs. Costs of these blood components were estimated using unit costs published in the 2022 to 2023 Annual Report of the Australian National Blood Authority.^16^ The costs associated with cryopreserved platelet manufacturing, logistics, and reconstitution were calculated with assistance from the Australian Red Cross Lifeblood (eTable 2 in Supplement 1).

### Health Outcome Measures

The following secondary health outcomes of the CLIP-II trial were included as effectiveness measures: the volume of postoperative bleeding (in milliliters) in the first 24 hours after ICU admission, the total volume of postoperative bleeding (in milliliters), 90-day mortality, a composite bleeding outcome based on the Bleeding Academic Research Consortium (BARC) criteria, and serious adverse events (SAEs). BARC type 4 bleeding is defined as the presence of any of the following: intracranial bleeding within 48 hours, reoperation after closure of sternotomy, transfusion of 5 or more units of whole blood or RBC within the 48 hours (intraoperative or postoperative), or chest tube output 2 or more L within a 24-hour period.^17^ Only SAEs reasonably suspected by the site principal investigator to be possibly, probably, or definitely related to the study treatment were included.

### Cost-Effectiveness Outcomes

The cost-effectiveness of cryopreserved platelets vs liquid-stored platelets was assessed by comparing the mean costs and health outcomes between treatment groups. Incremental cost-effectiveness ratios (ICERs) were calculated by dividing the difference in mean total per-patient costs between the cryopreserved platelets and liquid-stored platelets groups by the corresponding difference in health outcomes. The cost-effectiveness outcomes included the cost per 1 mL of postoperative bleeding avoided within the first 24 hours post-ICU admission, cost per 1 mL of total postoperative bleeding avoided, cost per 1% reduction in 90-day mortality, cost per 1% reduction in BARC4 bleeding, and cost per 1% reduction in SAEs.

### Statistical Analysis

All analyses were conducted using Stata statistical software version 18 (StataCorp). Statistical significance was 2-sided and set at 5%. Baseline characteristics, intraoperative bleeding, and fluid resuscitation were summarized as proportions for categorical variables and medians with IQRs for continuous variables, given their nonnormal distribution. Differences between study groups were reported with 95% CIs using a generalized linear model with an identity link and binomial family for categorical variables and median regression for continuous variables. However, comparisons of resource use, costs, and health outcomes were presented as means with SDs or as counts with percentages to ensure alignment with ICER calculations.

To assess uncertainty, probabilistic sensitivity analysis was performed using bootstrapping with 1000 iterations for both incremental costs (between-group differences in mean total per-patient costs) and incremental effectiveness (between-group differences in mean health outcomes). Both unadjusted and adjusted bootstrapping were conducted, with adjustments made for hospital site, EuroSCORE II risk score, and baseline imbalance (extracardiac arteriopathy), consistent with the primary outcome of the RCT. In adjusted models, mean differences with 95% CIs were estimated using generalized linear model, using a binomial family distribution for binary outcomes and a γ family distribution for continuous outcomes. Cost-effectiveness planes were used to visualize bootstrapping results where appropriate.

Additionally, subgroup analyses were performed to explore heterogeneity in cost-effectiveness based on surgery complexity, as the primary outcome did not differ across 3 prespecified subgroups: blood group compatibility, preoperative aspirin usage, and first transfusion location (operating room vs ICU). Surgeries were classified as complex if they met any of the following criteria: involvement of multiple cardiac valves; at least 1 valve procedure in addition to the coronary arteries, aortic root, or ventricular wall; procedures involving the aortic arch or descending aorta; procedures for infective endocarditis; or procedures affecting the pulmonary circulation.

## Results

### Baseline Characteristics

Among 202 trial participants (153 [75.7%] male, median [IQR] age 66 [57-74] years), 104 were randomized to the cryopreserved platelets group and 98 were randomized to liquid-stored platelets. Compared to the liquid-stored platelets group, the cryopreserved platelets group had a higher proportion of males (81 [78%] vs 72 [73%]) and were older (median [IQR] age, 66 [57-74] vs 64 [55-74] years). Overall, baseline characteristics were well balanced between the groups (Table 1).

**Table 1.** Baseline Characteristics of Study Participants

Characteristic	Participants, No.(%)	
	Cryopreserved platelets (n = 104)	Liquid-stored platelets (n = 98)
Age, median (IQR), y	66 (57-74)	64 (55-74)
Sex		
Male	81/104 (78)	72/98 (73)
Female	23/104 (22)	26/98 (27)
Blood group		
A	45/104 (43)	40/98 (41)
B	13/104 (13)	10/98 (10)
AB	1/104 (1)	3/98 (3)
O	45/104 (43)	45/98 (46)
Rh+	90/104 (86)	84/98 (86)
Platelets, median (IQR), ×10^3^/µL	199 (157-245)	204 (171-259)
aPTT, median (IQR), s	31 (29-34)	31 (29-34)
Fibrinogen, median (IQR), mgd/L	370 (300-460)	370 (300-450)
International normalized ratio, median (IQR)	1.1 (1-1.1)	1.1 (1-1.1)
Hemoglobin, median (IQR), g/dL	13.1 (11.7-14.5)	13.7 (12.2-14.7)
Elective surgery	73/104 (70)	68/98 (69)
Creatinine clearance >85 mL/min	40 (38)	41 (42)
Previous cardiac surgery	28/104 (27)	31/98 (32)
Chronic lung disease	15/104 (14)	11/98 (11)
Endocarditis	13/104 (12)	8/98 (8)
Cardiogenic shock	4/104 (4)	0/98 (0)
Extracardiac arteriopathy	9/104 (8.6)	1/98 (1)
NYHA classification of HF severity		
I	28/104 (27)	38/98 (39)
II	45/104 (43)	37/98 (38)
III	28/104 (27)	22/98 (22)
IV	3/104 (3)	1/98 (1)
Left ventricular function ≥51%	78/104 (76)	64/98 (66)
MI in the past 90 d	7/104 (7)	12/98 (12)
Pulmonary hypertension	28/104 (27)	26/98 (26)
EuroSCORE II risk score, median (IQR)	3 (2-8)	3/98 (2-6)
Anticoagulant therapy in the past 7 d	62/104 (60)	58/98 (59)
Tranexamic acid in the past 24 h	8/104 (8)	7/98 (7)

Abbreviations: aPTT, activated partial thromboplastin time; EuroSCORE II, European System for Cardiac Operative Risk Evaluation II; HF, heart failure; MI, myocardial infarction; NYHA, New York Heart Association.

SI conversion factors: To convert fibrinogen to grams per liter, multiply by 0.01; hemoglobin as grams per liter, multiply by 10; platelets to ×10^9^/L, multiply by 1.

### Resource Use and Costs

Participants in the cryopreserved platelets group remained in the ICU a mean of 1.6 (95% CI, 0.1 to 3.2) days longer and required 0.9 (95% CI, 0.5 to 1.2) additional unit of platelets during the index ICU stay compared with those in the liquid-stored platelets group. While participants in the cryopreserved platelets group used more resources, including ICU readmissions and readmission LOS, RBC, fresh frozen plasma, and cryoprecipitate, these differences were not statistically significant. Conversely, a higher proportion of participants in the liquid-stored platelets group were rehospitalized and had a mean hospital LOS 3 (95% CI, −1 to 8) days longer than those in the cryopreserved platelets group, although these differences were also not statistically significant. Detailed resource use is provided in Table 2.

**Table 2.** Resource Utilization Up to 90 Days Postdischarge by Study Groups

Resource	Mean (SD)		Difference (95% CI)
	Cryopreserved platelets (n = 104)	Liquid-stored platelets (n = 98)	
Index hospital LOS, d	18.9 (16.3)	17.1 (13.3)	1.8 (−2.3 to 5.9)
During index ICU stay			
Index ICU LOS, d	5.9 (6.9)	4.3 (4.1)	1.6 (0.1 to 3.2)
Participants readmitted to ICU, No. (%)	7/104 (7)	3/98 (3)	4 (−2 to 10)
ICU readmission LOS, d	5.6 (3.6)	7.3 (1.5)	−1.7 (−6.1 to 2.6)
First platelet order-to-receipt time, min	81 (67)	81 (73)	0 (19 to 20)
Participants receiving, No. (%)			
3 study platelet units	21/104 (20)	18/98 (18)	2 (−9 to 13)
2 study platelet units	41/104 (40)	26/98 (27)	13 (0 to 26)
1 study platelet unit	42/104 (40)	54/98 (55)	−15 (−28 to −1)
Study platelets, units	1.8 (0.7)	1.6 (0.8)	0.2 (−0.1 to 0.4)
Nonstudy platelets, units	1.1 (1.9)	0.2 (0.7)	0.9 (0.5 to 1.2)
RBC, units	6.7 (6.4)	5.1 (5.7)	1.6 (−0.4 to 3.7)
FFP, units	4.7 (3.7)	4.2 (2.8)	0.5 (−0.6 to 1.6)
Cryoprecipitate, units	11.0 (7.3)	8.8 (7.2)	2.2 (−0.4 to 4.7)
Post-ICU to hospital discharge LOS, d	12.7 (12.1)	12.6 (11.8)	0.1 (−3.3 to 3.4)
Postdischarge to 90 d			
Rehospitalized participants, No. (%)	15/104 (14)	19/98 (19)	−5 (−15 to 5)
Rehospitalization LOS, d	4.2 (2.7)	7.2 (8.4)	−3 (−8 to 1)
Discharged to rehabilitation, No. (%)	10/93 (10.7)	11/94 (11.7)	−1 (−10 to 8)

Abbreviations: FFP, fresh frozen plasma; ICU, intensive care unit; LOS, length of stay; RBC, red blood cells.

The differences in resource use were reflected in mean per-patient costs. The total mean per-patient cost in the cryopreserved platelets group was A$81 029 (A$72 395), with a mean difference of A$15 035 (95% CI, −A$1878 to A$31 949) in the cryopreserved platelets group vs liquid-stored platelets group (Table 3). Mean per-patient costs were nearly 50% higher for index ICU stay and 8 times higher for cryopreserved platelets transfusion compared with those in the liquid-stored platelets group. With the exception of index hospitalization and ICU readmissions, the cryopreserved platelets group incurred significantly higher mean per-patient costs compared with the liquid-stored platelets group.

**Table 3.** Per-Patient Costs in and Health Outcomes by Study Group

Measure	Mean (SD)		Difference (95% CI)
	Cryopreserved platelets (n = 104)	Liquid-stored platelets (n = 98)	
**Costs, A$**			
Index ICU stay	36 615 (51 211)	24 803 (27 030)	11 812 (636 to 22 998)
Index hospitalization	31 646 (30 383)	31 493 (29 509)	153 (−8086 to 8393)
ICU readmissions	2297 (10 105)	1375 (7893)	922 (−1565 to 3408)
Rehospitalizations	1514 (4479)	3494 (11 565)	−1980 (−4419 to 460)
Rehabilitation	1892 (5828)	2208 (6243)	−316 (−1980 to 1347)
Study platelets	3211 (1349)	400 (191)	2811 (2550 to 3073)
Nonstudy platelets	266 (463)	53 (162)	213 (119 to 308)
Red blood cells	1775 (2220)	1071 (1809)	704 (148 to 1259)
Fresh frozen plasma	539 (575)	323 (443)	216 (76 to 357)
Cryoprecipitate	1274 (1294)	775 (1123)	499 (166 to 832)
Total	81 029 (72 395)	65 993 (48 940)	15 035 (−1878 to 31 949)
**Health outcomes**			
Blood loss, mL^a^			
Within 24 h post-ICU admission	736 (491)	615 (341)	121 (5 to 237)
Total postoperative	1724 (1614)	1220 (905)	504 (145 to 862)
BARC4 bleeding, No. (%)	33/104 (31.7)	19/98 (19.4)	12.3 (1 to 23.6)
Serious adverse events, No. (%)	1/104 (1)	1/98 (1)	0 (−2.7 to 2.7)
Mortality at 90 d, No. (%)	13/104 (12.5)	5/98 (5.1)	7.4 (−0.3 to 15.1)

Abbreviations: BARC4, type 4 bleeding according to the Bleeding Academic Research Consortium; ICU, intensive care unit.

Conversion factor: To convert Australian dollars (A$) to US dollars, multiply by 0.73.

^a^ Excludes one participant from the cryopreserved group due to a missing value.

### Health Outcome Summaries

Participants in the cryopreserved platelets group had a mean (SD) blood loss of 736 (491) mL within 24 hours post-ICU admission, which was 121 (95% CI, 5 to 237) mL higher than the liquid-stored platelets group. Similarly, the mean (SD) total postoperative blood loss was 1724 (1614) mL in the cryopreserved platelets group, 504 (95% CI, 145 to 862) mL higher than in the liquid-stored platelets group. Additionally, 33 patients (31.7%) in the cryopreserved platelets group experienced BARC4 bleeding, compared with 19 patients (19.4%) in the liquid-stored platelets group. Only 1 SAE was observed in each group. The mortality rate during the study follow-up was 12.5% in the cryopreserved platelets group, with a difference of 7.4% (95% CI, −0.3% to 15.1%) compared with the liquid-stored platelets group. Further details are presented in Table 3.

### Cost-Effectiveness Summaries

In both unadjusted and adjusted bootstrapping analyses, cryopreserved platelets were dominated by liquid-stored platelets across all cost-effectiveness outcomes, indicating that the cryopreserved platelets group had higher costs and worse health outcomes relative to the liquid-stored platelets group (Table 4). This dominance is evident on cost-effectiveness planes for all cost-effectiveness outcomes (Figure; eFigures 1-5 in Supplement 1).

**Table 4.** Summary of Cost-Effectiveness Outcomes

Outcome	Incremental cost (95% CI), A$	Incremental effectiveness (95% CI)	ICER (95% CI)
**Unadjusted**			
Cost per 1 mL of bleeding avoided within 24 h ICU admission^a^	15 193 (−1647 to 33 546)	−119 (−238 to −2)^b^	Dominated^c^
Cost per 1 mL of total postoperative bleeding avoided	15 193 (−1647 to 33 546)	−494 (−860 to −159 )^b^	Dominated^c^
Cost per 1% reduction in BARC4 bleeding	14 567 (−2355 to 32 417)	−11.8 (−24.2 to −0.2)^d^	Dominated^c^
Cost per 1% reduction in 90-d mortality	14 567 (−2355 to 32 417)	−7.4 (−15.1 to −0.2)^d^	Dominated^c^
**Adjusted for EuroSCORE II risk score, site, and baseline imbalance (extracardiac arteriopathy)**			
Cost per 1 mL of bleeding avoided within 24 h ICU admission^a^	12 791 (−758 to 25 935)	−91 (−219 to 24)^b^	Dominated^c^
Cost per 1 mL of total postoperative bleeding avoided	12 791 (−758 to 25 935)	−324 (−605 to −55)^b^	Dominated^c^
Cost per 1% reduction in BARC4 bleeding	12 738 (−772 to 27 124)	−12.2 (23.5 to −0.9)^d^	Dominated^c^
Cost per 1% reduction in 90-d mortality	12 738 (−772 to 27 124)	−6.6 (−15.1 to 1.8)^d^	Dominated^c^

Abbreviations: BARC4, type 4 bleeding according to the Bleeding Academic Research Consortium; EuroSCORE II, European System for Cardiac Operative Risk Evaluation II; ICER, incremental cost-effectiveness ratio; ICU, intensive care unit.

Conversion factor: To convert Australian dollars (A$) to US dollars, multiply by 0.73.

^a^ Excludes 1 participant from the cryopreserved group due to a missing value.

^b^ Expressed as milliliters.

^c^ Dominated indicates that the mean cost was higher and the health outcome was worse in the cryopreserved platelet group compared with the liquid-stored platelet group.

^d^ Expressed as percentage.

**Figure.**
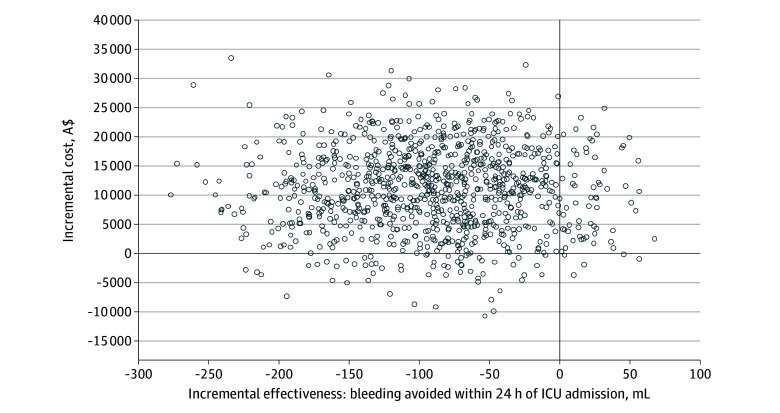
Cost-Effectiveness Plane of Incremental Costs and Incremental Effectiveness for Cryopreserved Platelets vs Liquid-Stored Platelets Incremental effectiveness was measured as bleeding avoided within 24 hours of intensive care unit (ICU) admission. To convert Australian dollars (A$) to US dollars, multiply by 0.73.

### Subgroup Analyses

Across all cost-effectiveness outcomes, cryopreserved platelets were dominated by liquid-stored platelets, regardless of cardiac surgery complexity (eTable 3 in Supplement 1). As the subgroup analysis findings were consistent with the main analysis, cost-effectiveness planes were not generated.

## Discussion

This cost-effectiveness study found that treatment with cryopreserved platelets was more costly and less effective than standard therapy with liquid-stored platelets for managing active bleeding in patients undergoing cardiac surgery at metropolitan Australian hospitals. The higher mean per-patient costs of cryopreserved platelets were primarily driven by longer ICU stays and a 7-fold increase in the costs of cryopreserved platelets utilization, including manufacturing and transportation of frozen platelets. Additionally, cryopreserved platelets were associated with greater bleeding volumes and higher mortality rates. In this economic evaluation, we provide novel evidence supporting the dominance of liquid-stored platelets over cryopreserved platelets in postoperative bleeding management following cardiac surgery.

To our knowledge, no previous cost-effectiveness analyses have compared cryopreserved platelets with liquid-stored platelets, and this study provides novel evidence on differences in health outcomes and costs between these products. The observed mean difference in 24-hour blood loss did not reach the clinically meaningful threshold defined in the CLIP-II trial (20% of the anticipated primary outcome blood loss, or approximately 1 unit of RBC transfused, equivalent to 258 mL in Australia). However, the greater total postoperative blood loss and higher rates of BARC4 bleeding in the cryopreserved platelets group were consistent with the longer ICU and hospital stays observed. It is well established that bleeding and blood transfusion in patients undergoing cardiac surgery lead to increased health care resource use and hospital costs. In 2 large US studies including a total of 182 800 patients undergoing cardiac surgery, those who experienced bleeding or received a blood transfusion had longer hospital and ICU stays and higher hospitalization costs compared with those without bleeding or transfusion.^18,19^ Similar findings were reported in studies conducted in Australia,^20^ Germany,^21^ and England,^22^ where patients with higher bleeding volumes had greater hospitalization costs. Our study confirms the association of increased hospital expenditure with bleeding volume and blood transfusion use and contributes new evidence to the limited economic literature on cryopreserved platelets by presenting their associated costs.

Platelet storage methods and duration may affect transfusion safety and effectiveness. A systematic review of 23 studies found that older platelets, compared with fresh platelets, were associated with a higher risk of transfusion reactions (relative risk [RR], 1.53 [95% CI, 1.04-2.25]), shorter transfusion intervals (RR, 0.25 [95% CI, 0.13-0.38] days), and increased platelet use.^23^ There was no significant difference in bleeding risk (RR, 1.13 [95% CI, 0.97-1.32]), and no significant difference in mortality was observed (RR, 1.03 [95% CI, 0.86-1.24]). In our study, cryopreserved platelets were frozen on day 2 after collection,^5^ stored for 12 months,^24^ and transfused within 4 hours of thawing.^25^ This process may have limited their effectiveness. Given these potential limitations and the minimal platelet wastage in metropolitan areas,^26^ routine use of cryopreservation in these settings may not be warranted.

Cryopreservation offers a viable solution for regional and remote areas where access to platelets is limited by low demand, transport constraints, and high waste associated with the 5- to 7-day shelf life of liquid-stored platelets. Platelet use in Australia has nearly doubled over 2 decades,^16,27^ and 80% of requests require supply within 24 hours,^26^ highlighting the urgency of timely availability. Although our study found that cryopreserved platelets were dominated by liquid-stored platelets in metropolitan cardiac surgery settings, this does not preclude their potential value in nonmetropolitan hospitals. In many regional and remote locations, liquid-stored platelets are often unavailable, making the relevant comparator no platelets at all rather than liquid-stored platelets. In such settings, cryopreserved platelets may improve equity by providing access to transfusion support that would otherwise be inaccessible. Further evaluation is warranted to assess the potential costs and health benefits of cryopreserved platelet implementation for emergency bleeding management in nonmetropolitan service environments.

### Strengths and Limitations

There are several notable strengths and limitations of this cost-effectiveness analysis. Key strengths include the rigorous trial design, which ensured that differences in costs and outcomes were directly attributable to the intervention, and the use of prespecified patient-level health care resource utilization data, enabling accurate cost assessment.

Nonetheless, several limitations should be considered when interpreting the findings. The strict inclusion and exclusion criteria of the RCT produced a relatively homogeneous study population, which may not fully represent patients undergoing cardiac surgery in clinical practice. Additionally, because resource use was collected from 11 high-resource metropolitan hospitals, external validity may be limited, as cost structures and clinical practices in regional and remote settings may differ. ICU cost weights by organ support were derived from UK National Cost Collection data due to the lack of Australian equivalents; differences in case-mix, practice patterns, and resource use between these countries may limit the precision of these estimates.

Readmission outcomes may also have been affected by informative censoring, as higher mortality and longer index admissions in the cryopreserved platelets group reduced the time at risk for subsequent rehospitalization. Therefore, rehospitalization rates and readmission LOS should be interpreted with caution. Furthermore, the economic evaluation did not account for societal costs, such as productivity loss, which could influence the overall cost-effectiveness of cryopreserved platelets. While the RCT was sufficiently powered to detect clinical differences in postoperative bleeding within 24 hours post-ICU admission among 202 participants, it was not powered to detect cost differences, as cost data tend to be highly variable and often require larger sample sizes than clinical outcomes. Furthermore, the cost of cryopreserved platelets used in the trial may be overestimated, as production costs would likely decrease if cryopreserved platelets were manufactured routinely, rather than as a clinical trial product.

## Conclusions

This economic evaluation found that among patients undergoing cardiac surgery in metropolitan hospitals in Australia, standard therapy with liquid-stored platelets was more effective and cost-efficient than cryopreserved platelets for managing active bleeding. However, further evaluation of the potential costs and benefits of cryopreserved platelets implementation in regional and remote settings may be warranted to ensure equitable access to emergency bleeding management across the country.

## Notes

Supplement 1. eTable 1. Unit costs of resource utilisation for the CLIP-II trial eTable 2. Components of cryopreserved platelet costs per unit obtained from the Australian Red Cross Lifeblood eTable 3. Subgroup analyses by complexity of cardiac surgeries eFigure 1. Cost-effectiveness plane of incremental costs and total postoperative bleeding for CPP vs LSP (adjusted) eFigure 2. Cost-effectiveness plane incremental costs and incremental effectiveness (24-hour bleeding for CPP vs LSP (unadjusted) eFigure 3. Cost-effectiveness plane incremental costs and total postoperative bleeding for CPP vs LSP (unadjusted) eFigure 4. Cost-effectiveness plane of incremental costs and incremental effectiveness (BARC4 bleeding) for CPP vs LSP eFigure 5. Cost-effectiveness plane of incremental costs and incremental effectiveness (90-day mortality) for CPP vs LSP eReferences.

Supplement 2. Data Sharing Statement
