# Supplementary material for: Cost-Effectiveness of Cryopreserved vs Liquid-Stored Platelets for Managing Surgical Bleeding
Source: JAMA Netw Open. 2025 Dec 8;8(12):e2554363. doi: 10.1001/jamanetworkopen.2025.54363 (PMC12687093; doi:10.1001/jamanetworkopen.2025.54363)
Supplement: Supplement 1. — eTable 1. Unit costs of resource utilisation for the CLIP-II trial eTable 2. Components of cryopreserved platelet costs per unit obtained from the Australian Red Cross Lifeblood eTable 3. Subgroup analyses by complexity of cardiac surgeries eFigure 1. Cost-effectiveness plane of incremental costs and total postoperative bleeding for CPP vs LSP (adjusted) eFigure 2. Cost-effectiveness plane incremental costs and incremental effectiveness (24-hour bleeding for CPP vs LSP (unadjusted) eFigure 3. Cost-effectiveness plane incremental costs and total postoperative bleeding for CPP vs LSP (unadjusted) eFigure 4. Cost-effectiveness plane of incremental costs and incremental effectiveness (BARC4 bleeding) for CPP vs LSP eFigure 5. Cost-effectiveness plane of incremental costs and incremental effectiveness (90-day mortality) for CPP vs LSP eReferences. [file jamanetwopen-e2554363-s001.pdf]

Supplemental Online Content

Orman Z, Reade MC, Marks DC, et al. Cost-effectiveness of cryopreserved platelets vs liquid-stored platelets for managing surgical bleeding. *JAMA Netw Open*. 2025;8(12):e2554363. doi:10.1001/jamanetworkopen.2025.54363

eTable 1. Unit costs of resource utilisation for the CLIP-II trial

eTable 2. Components of cryopreserved platelet costs per unit obtained from the Australian Red Cross Lifeblood

eTable 3. Subgroup analyses by complexity of cardiac surgeries

eFigure 1. Cost-effectiveness plane of incremental cost and incremental effectiveness (total postoperative bleeding) for cryopreserved vs liquid-stored platelets (adjusted)

eFigure 2. Cost-effectiveness plane incremental cost and incremental effectiveness (24-hour bleeding) for cryopreserved vs liquid-stored platelets (unadjusted)

eFigure 3. Cost-effectiveness plane incremental cost and total postoperative bleeding for cryopreserved vs liquid-stored platelets (unadjusted)

eFigure 4. Cost-effectiveness plane of incremental cost and incremental effectiveness (BARC4 bleeding) for cryopreserved vs liquid-stored platelets

eFigure 5. Cost-effectiveness plane of incremental cost and incremental effectiveness (90-day mortality) for cryopreserved vs liquid-stored platelets

eReferences.

This supplemental material has been provided by the authors to give readers additional information about their work.

© 2025 Orman Z et al. *JAMA Network Open*.

eTable 1. Unit costs of resource utilisation for the CLIP-II trial

Resources	Cost per unit, 2023 A$
Index ICU stay per bed-day based on the number of organs supported each day:[1,2]	
Adult Critical Care, 0 Organs Supported	3002
Adult Critical Care, 1 Organ Supported	4273
Adult Critical Care, 2 Organ Supported	5646
Adult Critical Care, 3 Organ Supported	7013
Adult Critical Care, 4 Organ Supported	9112
Adult Critical Care, 5 Organ Supported	10 684
Adult Critical Care, 6 or more Organ Supported	13 518
Subsequent ICU stay per bed-day[2,3]	6125
Hospitalisation per bed-day[3,4]	2499
Rehabilitation per case[3,5]	19 675
Platelet transfusion per unit (whole blood)[6]	245
Red blood cells per unit (not washed)[6]	357
Fresh frozen plasma per unit(whole blood)[6]	152
Cryoprecipitate transfusion per unit (whole blood)[6]	163
Cryopreserved platelets per unit (Marks D, unpublished data, 2025)*	1786

*Obtained from the Australian Red Cross.

Abbreviations: ICU, intensive care unit.

Conversion factor: To convert Australian dollars (A$) to US dollars, multiply by 0.73.

© 2025 Orman Z et al. *JAMA Network Open*.

eTable 2. Components of cryopreserved platelet costs per unit obtained from the Australian Red Cross Lifeblood

Resources	Cost per unit, 2023 A$ (%)
Cryopreserved platelets per unit:	1786 (100)
Freezing expenses	1436/1786 (80)
Workload expenses	147/1786 (8)
Shipping expenses	86/1786 (5)
Environmental monitoring expenses	51/1786 (3)
Quality control expenses	36/1786 (2)
Equipment maintenance expenses	30/1786 (2)

Conversion factor: To convert Australian dollars (A$) to US dollars, multiply by 0.73.

© 2025 Orman Z et al. *JAMA Network Open*.

eTable 3. Sub-group analyses by complexity of cardiac surgeries

Outcome	Incremental cost (95% CI), A$	Incremental effectiveness (95% CI)	ICER (95% CI)
Complex surgeries[a]			
Cost per 1 mL of bleeding avoided within 24 h ICU admission	17 335 (-4850 to 43 051)	-101 (-248; 43)[b]	Dominated[c]
Cost per 1 mL of total post-operative bleeding avoided	17 335 (-4850 to 43 051)	-448 (-962; 9)[b]	Dominated[c]
Cost per 1% reduction in BARC4 bleeding	17 335 (-4850 to 43 051)	-14.6 (-29.4; 0.7)[d]	Dominated[c]
Cost per 1% reduction in 90-day mortality	17 335 (-4850 to 43 051)	-6.5 (-16.9; 3.8)[d]	Dominated[c]
Non-complex surgeries[a]			
Cost per 1 mL of bleeding avoided within 24h ICU admission	7425 (-10 148 to 23 646)	-147 (-317; 45)[b]	Dominated[c]
Cost per 1 mL of total post-operative bleeding avoided	7425 (-10 148 to 23 646)	-552 (-1,073; -80)[b]	Dominated[c]
Cost per 1% reduction in BARC4 bleeding	7425 (-10 148 to 23 646)	-5.8 (-24.9; 11.5)[d]	Dominated[c]
Cost per 1% reduction in 90-day mortality	7425 (-10 148 to 23 646)	-8.7 (-21.4; 2.6)[d]	Dominated[c]

[a]Surgeries were classified as “complex” if they met any of the following criteria: involvement of multiple cardiac valves, at least one valve in addition to the coronary arteries, aortic root, or ventricular wall, procedures involving the aortic arch or descending aorta, surgeries for infective endocarditis, or those affecting the pulmonary circulation.
[b] Expressed as milliliters.
[c]Dominated indicates that the average cost is higher, and the health outcome is worse in the CPP group compared to the LSP group.
[d] Expressed as percentage.
Abbreviations: BARC4, type 4 bleeding according to the Bleeding Academic Research Consortium; CI, confidence interval; h, hours; ICU, intensive care unit; h, hours.

© 2025 Orman Z et al. *JAMA Network Open*.

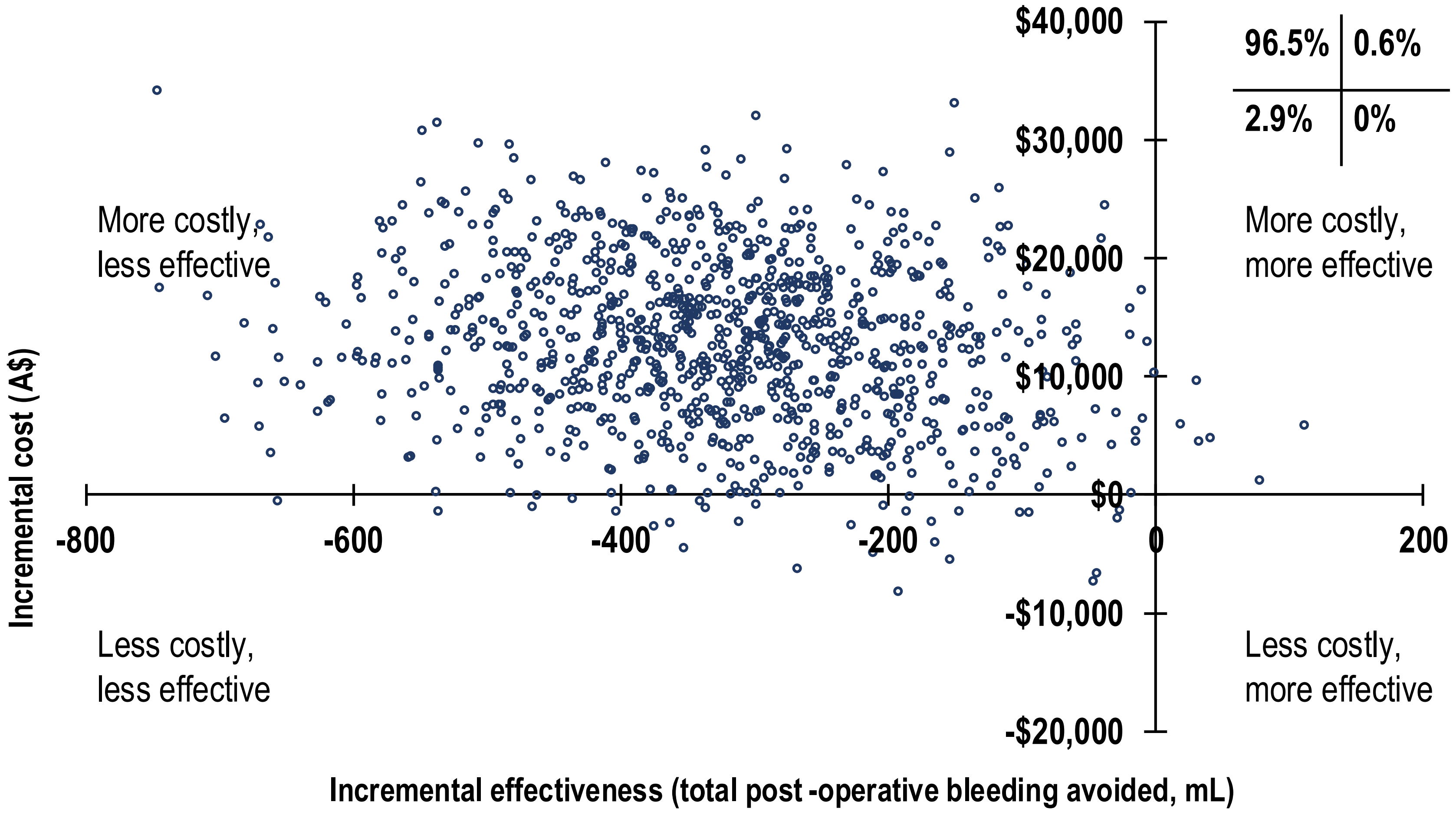

eFigure 1. Cost-effectiveness plane of incremental cost and incremental effectiveness (total postoperative bleeding) for cryopreserved vs liquid-stored platelets (adjusted)

To convert Australian dollars (A$) to US dollars, multiply by 0.73

.

© 2025 Orman Z et al. *JAMA Network Open*.

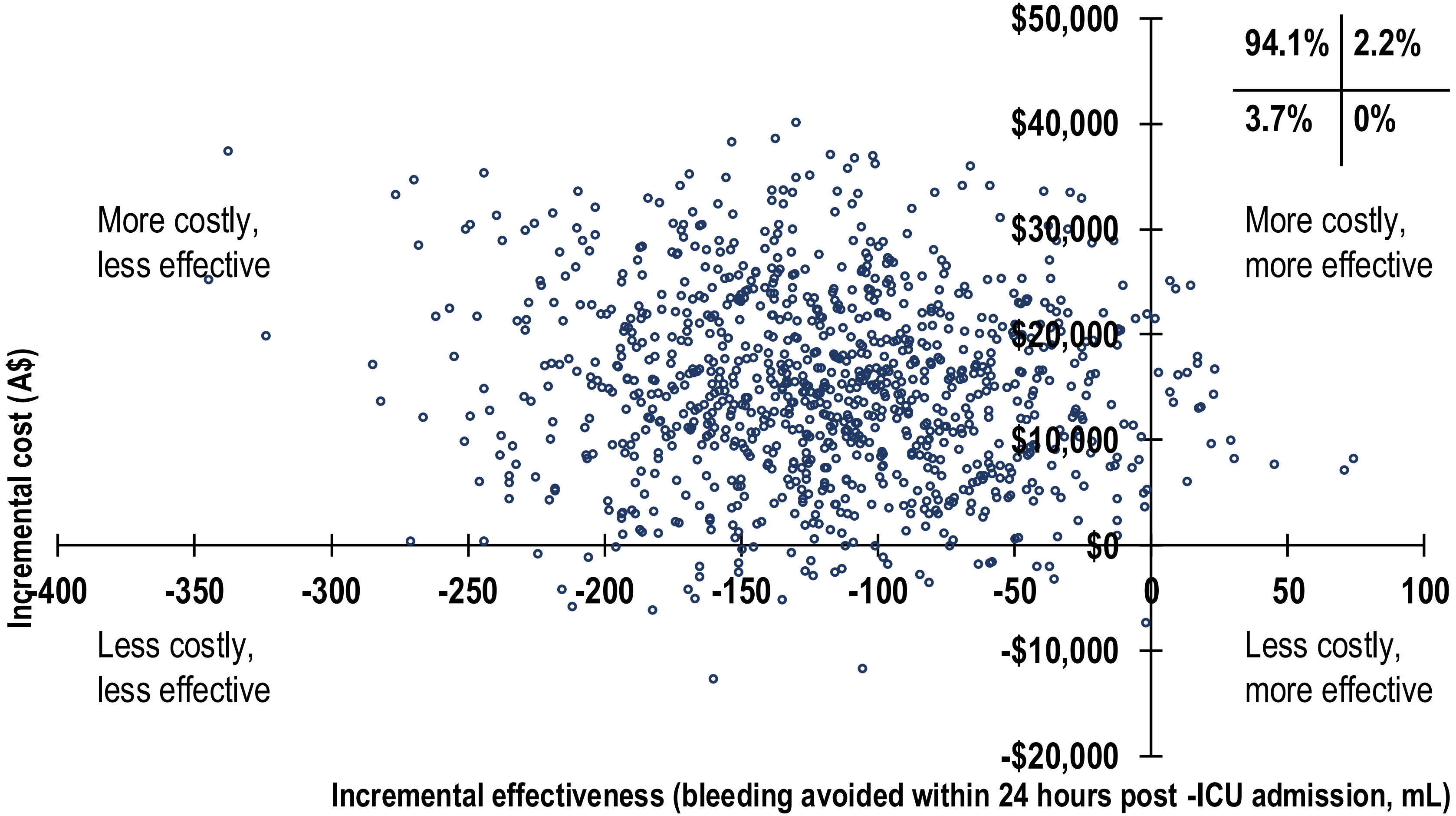

eFigure 2. Cost-effectiveness plane incremental cost and incremental effectiveness (24-hour bleeding for cryopreserved vs liquid-stored platelets (unadjusted)

To convert Australian dollars (A$) to US dollars, multiply by 0.73

© 2025 Orman Z et al. *JAMA Network Open*.

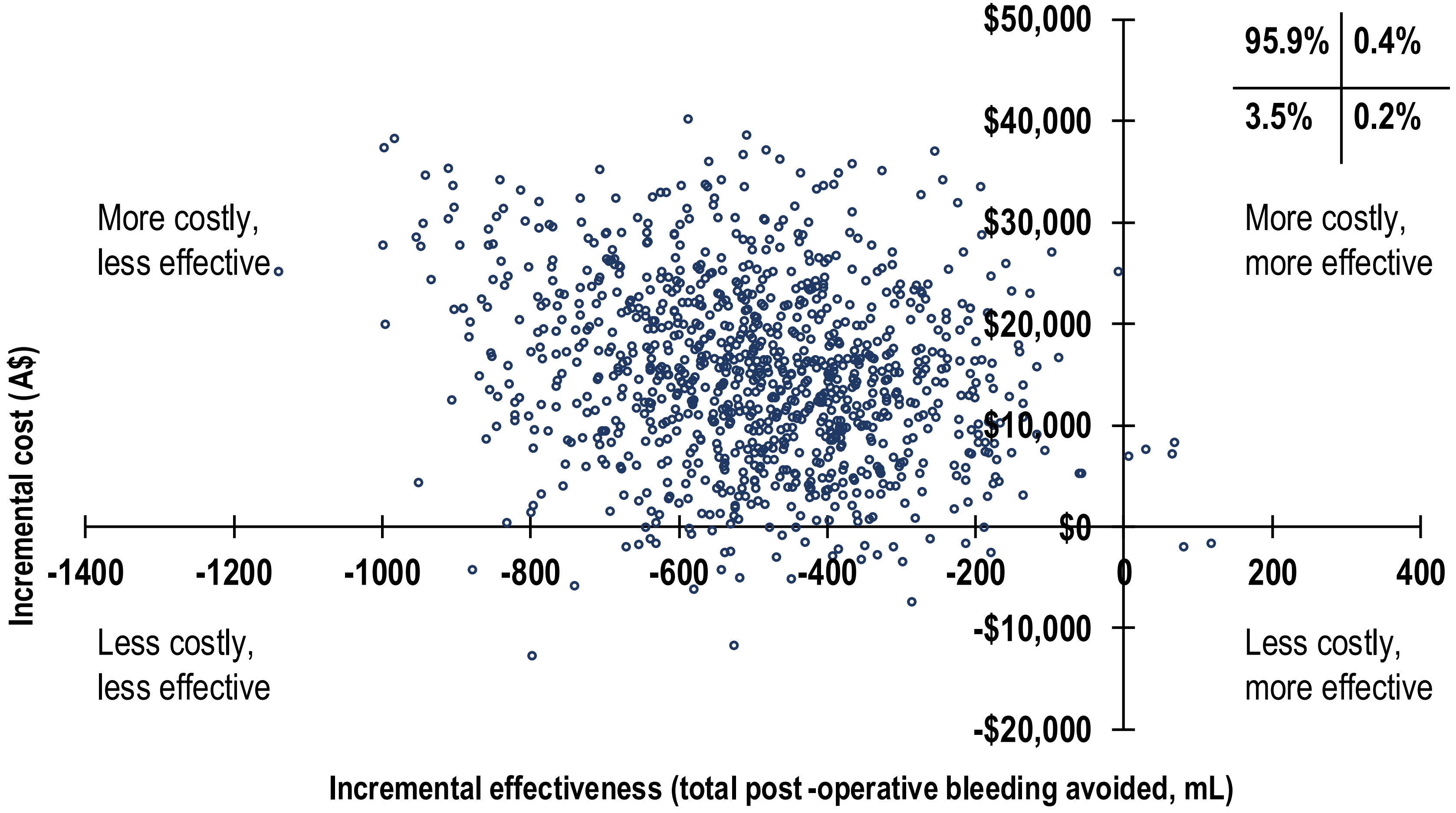

eFigure 3. Cost-effectiveness plane incremental cost and incremental effectiveness (total post-operative bleeding) for cryopreserved vs liquid-stored platelets (unadjusted)

To convert Australian dollars (A$) to US dollars, multiply by 0.73.

© 2025 Orman Z et al. *JAMA Network Open*.

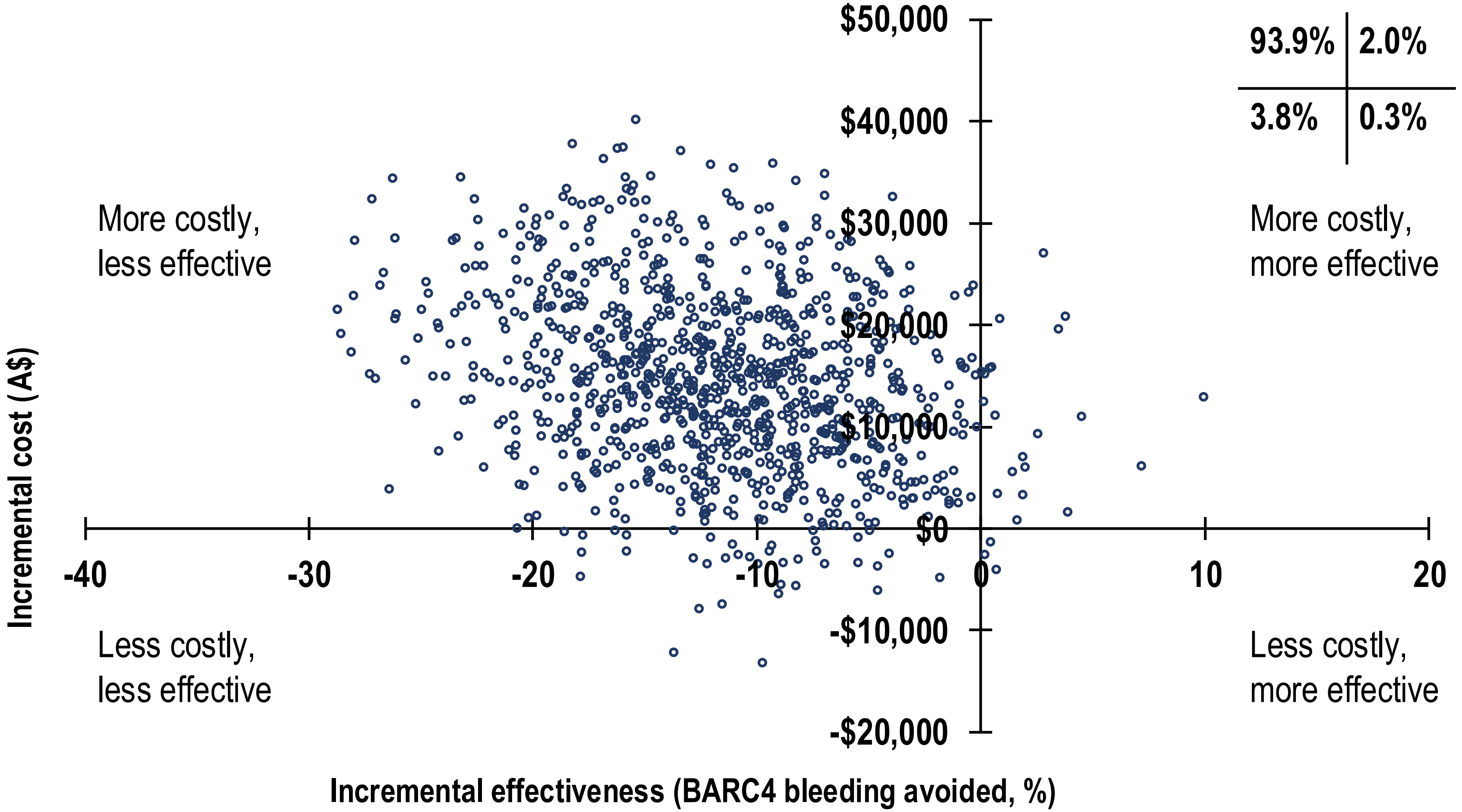

eFigure 4. Cost-effectiveness plane of incremental cost and incremental effectiveness (BARC4 bleeding) for cryopreserved vs liquid-stored platelets

To convert Australian dollars (A$) to US dollars, multiply by 0.73.

© 2025 Orman Z et al. *JAMA Network Open*.

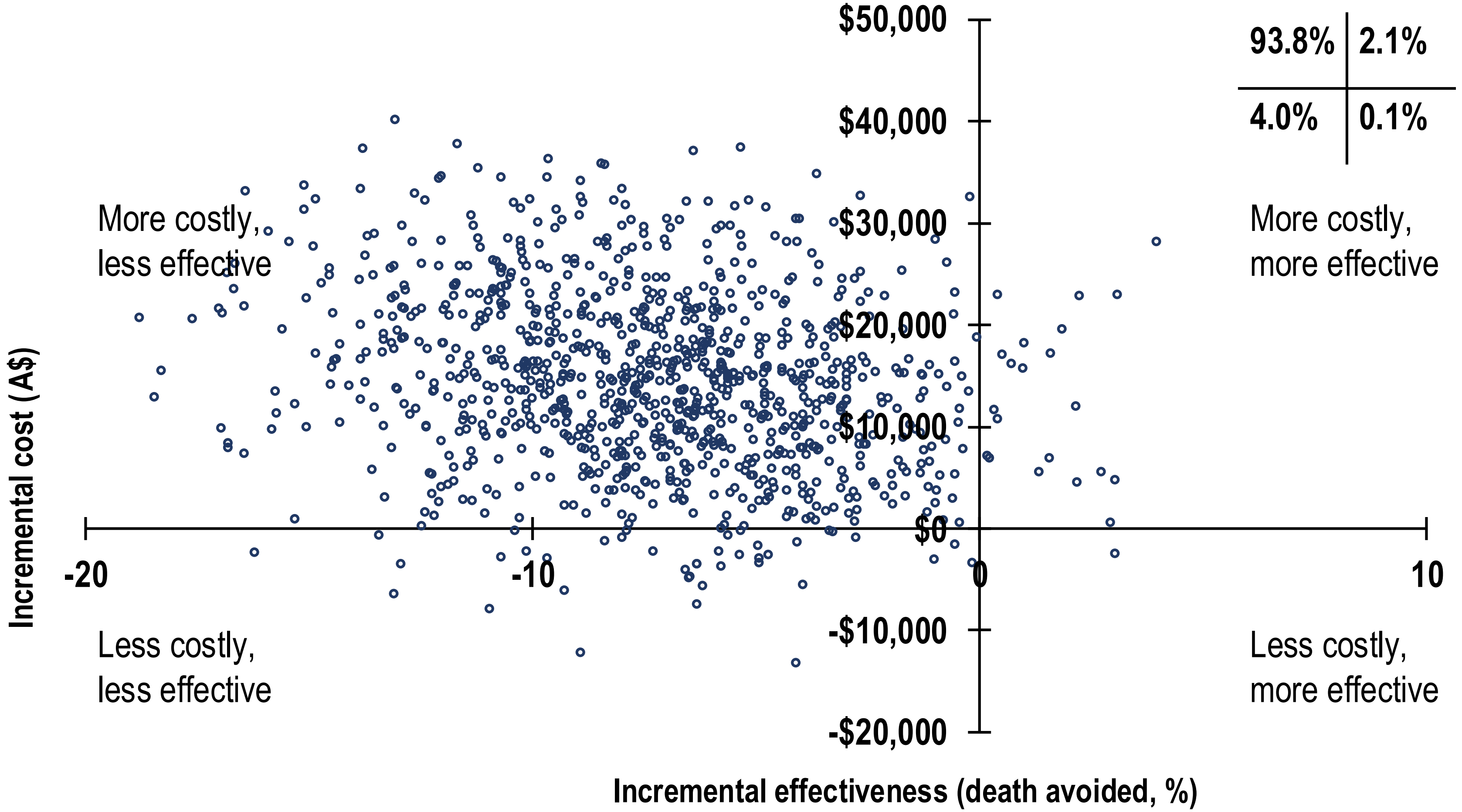

eFigure 5. Cost-effectiveness plane of incremental cost and incremental effectiveness (90-day mortality) for cryopreserved vs liquid-stored platelets

To convert Australian dollars (A$) to US dollars, multiply by 0.73.

© 2025 Orman Z et al. *JAMA Network Open*.

eReferences

1. National Health Service in England. National cost collection 2023 [Available from: https://www.england.nhs.uk/costing-in-the-nhs/national-cost-collection/. Accessed
2. Hicks P, Huckson S, Fenney E, Leggett I, Pilcher D, Litton E. The financial cost of intensive care in Australia: A multicentre registry study. *Med J Aust*. 2019;211(7):324–5.
3. Australian Bureau of Statistics. Consumer Price Index, Australia [internet]. Canberra: ABS; jun-quarter-2024 [cited 2024 october 8] 2024 [Available from: https://www.abs.gov.au/statistics/economy/price-indexes-and-inflation/consumer-price-index-australia/jun-quarter-2024. Accessed
4. Independent Hospital Pricing Authority. National Hospital Cost Data Collection: Public sector Report, 2021-22.
5. Independent Hospital Pricing Authority. Development of the australian national subacute and non-acute patient classification version 5.0. 2021.
6. National Blood Authority. Annual Report 2022-23.

© 2025 Orman Z et al. *JAMA Network Open*.
