# Supplementary material for: Cost-Effectiveness of Cryopreserved vs Liquid-Stored Platelets for Managing Surgical Bleeding
Source: JAMA Netw Open. 2025 Dec 8;8(12):e2554363. doi: 10.1001/jamanetworkopen.2025.54363 (PMC12687093; doi:10.1001/jamanetworkopen.2025.54363)
Supplement: Supplement 2. — Data Sharing Statement [file jamanetwopen-e2554363-s002.pdf]

Data Sharing Statement

Orman. Cost-Effectiveness of Cryopreserved vs Liquid-Stored Platelets for Managing Surgical Bleeding. *JAMA Netw Open*. Published December 08, 2025.
doi:10.1001/jamanetworkopen.2025.54363

Data
Data available: Yes
Data types: Deidentified participant data
How to access data: Requests for data can be made to lisa.higgins@monash.edu
When available: With publication

Supporting Documents
Document types: None

Additional Information
Who can access the data: Researchers whose proposed use of the data has been approved
Types of analyses: For a purpose reviewed and approved by the CLIP II management committee
Mechanisms of data availability: With a signed data access agreement
